# Mobile Device Applications for the Visualization of Functional Connectivity Networks and EEG Electrodes: iBraiN and iBraiNEEG

**DOI:** 10.3389/fninf.2016.00040

**Published:** 2016-10-19

**Authors:** Gonzalo M. Rojas, Jorge A. Fuentes, Marcelo Gálvez

**Affiliations:** ^1^Laboratory for Advanced Medical Image Processing, Department of Radiology, Clínica las CondesSantiago, Chile; ^2^Advanced Epilepsy Center, Clínica las CondesSantiago, Chile; ^3^Department of Radiology, Clínica las CondesSantiago, Chile

**Keywords:** functional connectivity, EEG, EEG-fMRI, fMRI, smartphone, mobile application, intrinsic connectivity networks

## Abstract

Multiple functional MRI (fMRI)-based functional connectivity networks were obtained by Yeo et al. (2011), and the visualization of these complex networks is a difficult task. Also, the combination of functional connectivity networks determined by fMRI with electroencephalography (EEG) data could be a very useful tool. Mobile devices are becoming increasingly common among users, and for this reason, we describe here two applications for Android and iOS mobile devices: one that shows in an interactive way the seven Yeo functional connectivity networks, and another application that shows the relative position of 10–20 EEG electrodes with Yeo’s seven functional connectivity networks.

## Introduction

Visualization of the human brain using complex images such as tractography, functional connectivity, functional imaging, brain volume, PET, SPECT, etc. is a significant challenge, and the fusion of such types of images produce an even more complex problem. Also, the increased use of functional connectivity based on functional MRI (fMRI) techniques, and the visualization of the complex networks obtained by this method, creates the need for advanced tools to visualize the various functional connectivity networks for academic purposes and research.

Functional connectivity is defined as the time dependence of neuronal activity between anatomically separate brain regions (Proal et al., 2011). There are multiple functional connectivity networks in a healthy brain. Yeo et al. (2011) with his network-level parcellation determined that there are at least seven standard functional connectivity networks in the healthy human brain. These are visual network, somatomotor, dorsal attention, ventral attention, limbic, frontoparietal and default network (default mode network, DMN). Examples of this are the (consisting of precuneus, medial frontal, inferior parietal cortical regions and medial temporal lobe), which is active during wakeful rest and deactivates during most externally oriented tasks (Raichle et al., 2001; Greicius, 2008; de la Iglesia-Vayá et al., 2013); and the somatomotor network related to sensitive and primary motor processing (de la Iglesia-Vayá et al., 2013).

Different neuroimaging visualization techniques have been proposed. For example, Margulies et al. (2013) describe 2-D and 3-D methods to display anatomical information, tractography and functional connectivity. Rojas et al. (2014) describe a method for displaying stereoscopic 3-D neuroimages by using red-cyan colors. Rojas et al. (2014), show examples of tractography, brain volumetry, and quantification of multiple sclerosis lesions and images of functional connectivity (network visualization).

Another brain test is Electroencephalography (EEG), a well-known electrophysiological diagnosis method that measures the electrical activity of the brain. It is a noninvasive technique in which electrodes are attached over the scalp. There are two systems for the placement of electrodes: 10–20 corresponding to 21 electrodes (Jasper, 1958; Klem et al., 1999), and 10–10 which corresponds to 65 electrodes (Nuwer et al., 1998). EEG is the principal diagnostic tool for diseases such as epilepsy.

Mobile devices (such as smartphones and tablets) are becoming increasingly common among users. In a study published by Scott Wilson on November 6, 2013 (Wilson, 2013), sales of phones and tablets are increasing in an annual growth rate of 21.8% (Table 1), implying a broad base of potential users for each application. Mainly, there are four operating systems for mobile devices: iOS used by Apple devices (13.9% of iPhone and iPad), Android used by Samsung Electronics Co Ltd., LG Electronics Inc., Sony Corporation, Google Inc., HTC Corporation, Motorola Inc., Huawei, Xiaomi, etc. (corresponding to 82.8%), Windows Phone (2.6%), Blackberry OS (0.3%) and others (0.4%; IDC Research, Inc., 2015). In the medical field, 79.0% of medical students and 74.8% of junior doctors in United Kingdom owned a smartphone (Payne et al., 2012), and in USA 87% of medical doctors use a smartphone or tablet device in their workplace (Ventola, 2014).

**Table 1:** **Worldwide annual sales growth of tablets and smartphones (millions of units; Wilson, 2013)**.

Device type	2011	2012	2013	2014	2015	2016	2017
Tablets	60	120	197	266	338	401	468
Smart phones	367	474	568	670	765	852	923
**Total**	427	594	765	936	1103	1253	1391

In this article, we describe two applications for iOS and Android-based mobile devices covering 96.7% of the mobile market. One application shows the seven standard functional connectivity networks (Yeo et al., 2011) superimposed on a cerebral cortex (transparent brain) and the other one interactive application shows 21 10–20 EEG electrodes over the Yeo et al. (2011) standard networks.

## Materials and Methods

### 3D Models (Mesh)

MNI152 image is a standard space T1-wieghted average structural template image. It was created by averaging 152 T1-weighted MRI images (normal young adults) linearly transformed to Talairach space (Mazziotta et al., 1995, 2001; Mandal et al., 2012).

Using the template image MNI152 (2 mm isotropic voxel size), a mesh model of the brain cortex was created using Grayscale Model Maker module (marching cubes algorithm [Lorensen and Cline, 1987]; grayscale threshold of isosurface: 5800, number of smoothing iterations: 50, target reduction during decimation: 25% triangles to be removed, split normal, calculate the normal vectors for the points, pad the input volume with zero value voxels) from the 3D Slicer 3.6.3 open-source software (Brigham and Women’s Hospital, Boston, MA, USA^1^, Gering et al., 1999; Pieper et al., 2004, 2006).

A mask of the network-level parcellation published by Yeo (Yeo et al., 2011,^2^) was used to create the mesh model of the seven standard functional networks (Yeo et al., 2011). These mesh models were built using the Model Maker module (marching cubes algorithm [Lorensen and Cline, 1987]; number of smoothing iterations: 10, filter type for smoothing: Sinc, target reduction during decimation: 25% triangles to be removed, split normals, calculate the normal vectors for the points, pad the input volume with zero value voxels) from the 3D Slicer 3.6.3 software.

The HC Laplacian algorithm (MeshLab v 1.3.3^3^, Vollmer et al., 1999; Cignoni et al., 2008a,b) was used to smoothen the 3D meshes of the seven Yeo networks and the brain cortex. Wavefront OBJ geometry definition file format was used to save the meshes.

### Design and Application Programming

The iOS and Android apps were created using C# programming language and the following software: Unity 4.6x (development engine to create 2D and 3D graphics applications^4^), MonoDevelop IDE^5^, Blender (3D graphics software^6^), GIMP (GNU Image manipulation program^7^). Xcode 6x Integrated Development Environment in MAC OSX (Apple Inc., Cupertino, CA, USA^8^) was used to create iOS-based version of both mobile applications.

MNI coordinates for standard 10–20 EEG electrodes were computed previously (Rojas and Gálvez, 2013). Then using that MNI coordinates, red spheres were positioned over the mesh of the brain cortex and the functional connectivity meshes.

## Results

Two applications called iBraiN2 and iBraiNEEG2 were developed for the visualization of Functional connectivity networks.

### iBraiN2

The application name, iBraiN2, is an acronym that stands for “intrinsic Brain Networks”.

**Figure 1:**
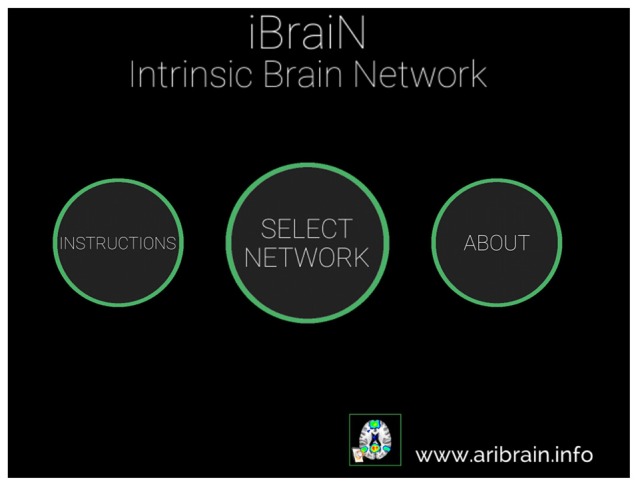
**Main window of the iBraiN2 application.** By touching “Instructions”, user instructions appear for iBraiN2, by pressing “About” iBraiN2 authoring information appears, and by touching the “Select Network” button the application displays a menu to select a functional connectivity network (see Figure 2).

**Figure 2:**
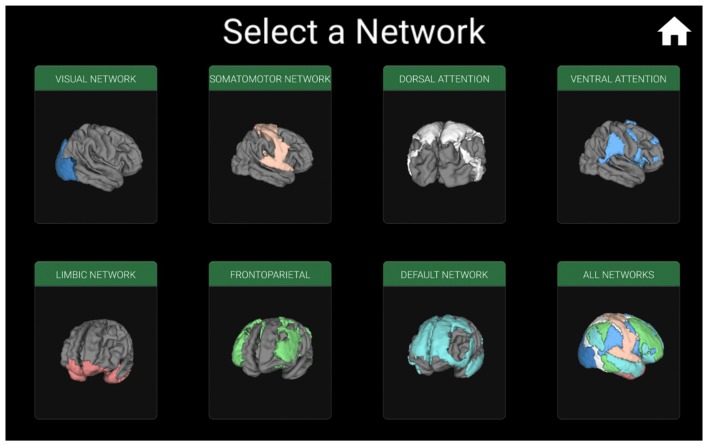
**Window for selecting the functional connectivity network which will be displayed individually, or all networks on a single brain.** For example, iBraiN2 will show the window in Figure 3 if the user selects “All Networks”, and the visual connectivity network (Yeo et al., 2011) from Figure 4 will appear if the user touches the “Visual Network” button.

**Figure 3:**
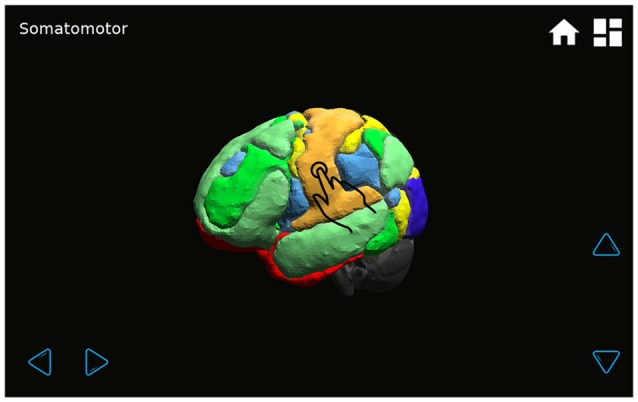
**A transparent brain with seven superimposed functional connectivity networks is shown by touching the “All Networks” button (Figure 2).** By pressing each network, its name will appear in the upper left-hand corner. The brain can be rotated by pressing the four light blue arrows.

**Figure 4:**
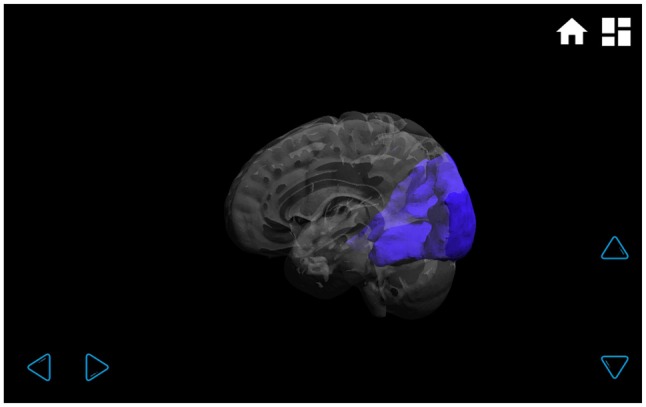
**Visual functional connectivity network.** In the transparent brain, it can be clearly seen that the visual network is located only in the occipital lobe, and it has no connectivity with other brain regions. The network can be rotated by touching the arrows on the lower right and lower left-hand corners.

**Figure 5:**
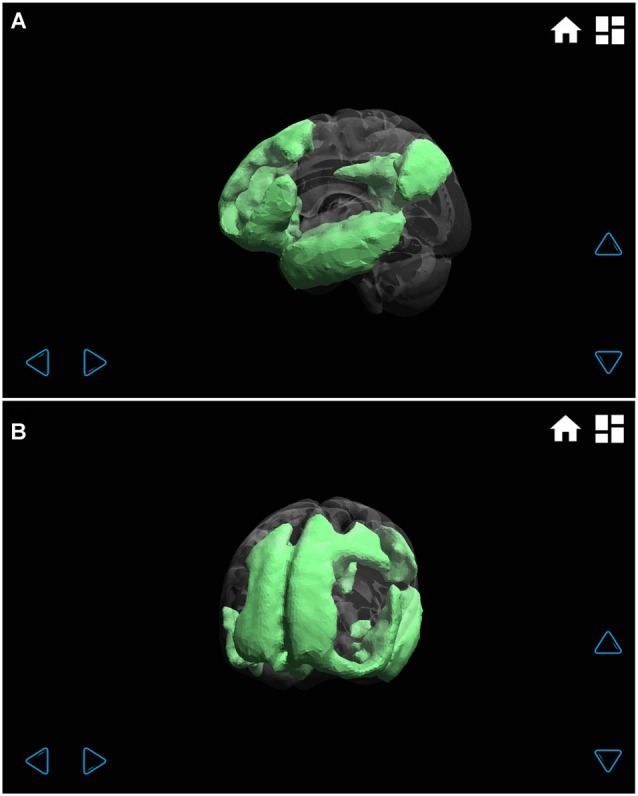
**Default mode functional connectivity network displayed at different angles (A left hemisphere, and B left frontal).** Through the transparent brain, the different regions that make up this wide network can be clearly seen: mainly frontal, parietal and temporal lobe regions.

The iBraiN2 application shows a 3-D transparent brain, merged with one of seven standard functional networks (Yeo et al., 2011) that can be selected by the user. The brain can be rotated using the controls on the screen (arrows), and the brain size can be changed by a pinch gesture. From Figures 1–5 the user can see the Graphical User Interface (GUI) of the application and how it is used.

**Figure 6:**
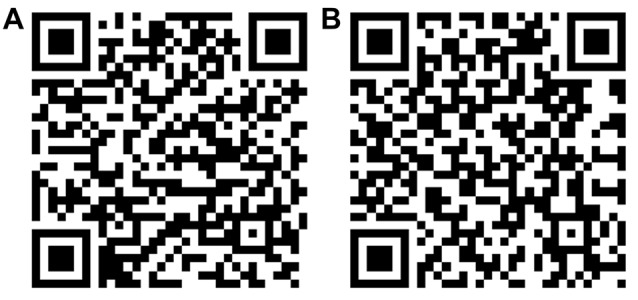
**QR code to install iBraiN application in **(A)** Android-based devices, **(B)** iOS-based devices**.

To install this app on an Android mobile device, users must select Google Play Store (Google Inc.) and search for the app by its name “iBraiN2” (to install it, use QR code in Figure 6A). In iOS, users must enter the App Store (Apple Inc.) and search for “iBraiN2” (to install it use QR code in Figure 6B).

### iBraiNEEG2

The application name, iBraiNEEG2, is an acronym standing for “intrinsic Brain Networks EEG”.

**Figure 7:**
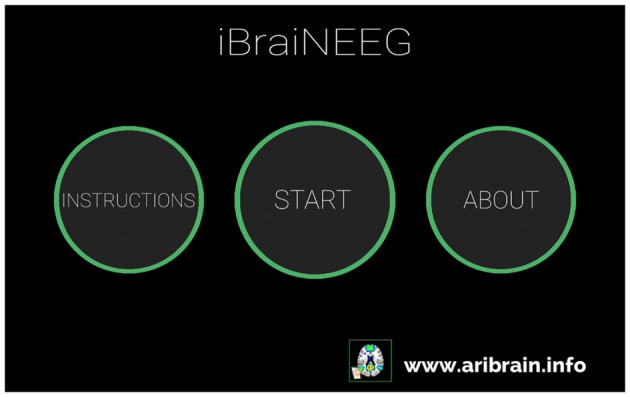
**iBraiNEEG2 main window.** iBraiNEEG2 user instructions will be shown by touching the “Instructions” button; by pressing “About” the iBraiNEEG2 authoring information appears, and “Start” shows the transparent brain with the seven connectivity networks (Yeo et al., 2011) and red spheres for the 10–20 electroencephalography (EEG) electrodes (Figure 8).

**Figure 8:**
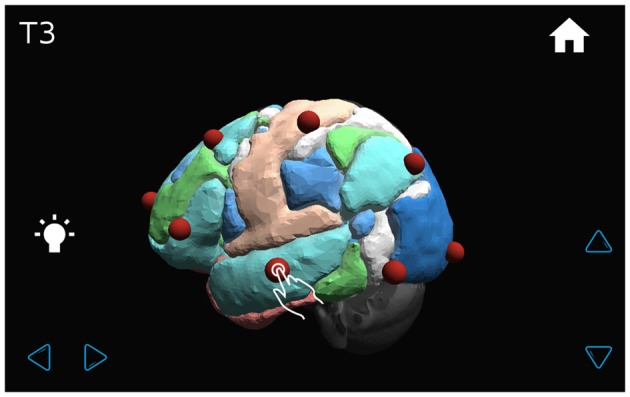
**The transparent brain is shown with seven connectivity networks (Yeo et al., 2011) and red spheres on the 10–20 EEG electrode positions.** By touching a sphere, the name of the electrode appears on the upper left-hand corner of the screen (for example, T3).

**Figure 9:**
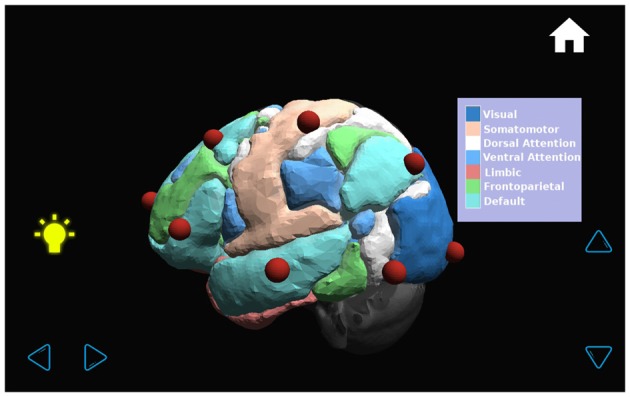
**By pressing the bulb on the left of the screen, the name of each of the seven functional connectivity networks (Yeo et al., 2011) appears with a color palette in a window on the right side of the screen**.

The application shows a transparent brain with seven connectivity networks upon it (Yeo et al., 2011) and 21 red spheres on the brain in the standard 10–20 EEG electrode positions as determined in a previous work (Rojas and Gálvez, 2013). The various GUI windows are shown in Figures 7–9. The name of each EEG electrode and functional network is displayed interactively by touching on each red sphere and on the light bulb, respectively Figures 8, 9.

The application shows 10–20 EEG electrode positions (21 electrodes) because it is difficult for the application user to select on the touchscreen more than 21 electrodes (65 electrodes of 10–10 EEG standard), and also because in clinical use the 10–20 EEG system is the most often used.

**Figure 10:**
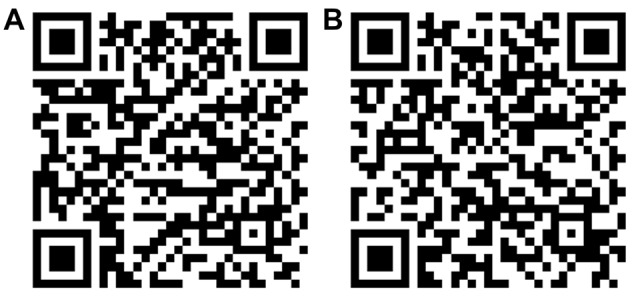
**QR code to install iBraiNEEG application in (A) Android-based devices, (B) iOS-based devices**.

To install this application on an Android mobile device, select Google Play Store (Google Inc.) and search for the “iBraiNEEG2” application (use QR code in Figure 10A). In iOS App Store (Apple Inc.), search “iBraiNEEG” (without the number 2) and install (to install it use QR in Figure 10B). More information about both applications can be found at http://www.aribrain.info website.

## Discussion

Both applications show the advanced uses that iOS and Android-based mobile devices (tablets and smartphones) can be given in the medical field. As far as we know, iBraiN2 and iBraiNEEG2 are the first functional connectivity applications for iOS/Android-based mobile devices. These applications are different from previous work: (i) the atlas in Rojas et al. (2014), which is a functional connectivity anaglyph, based in area-level parcellation (Craddock et al., 2012) for visualization in PC with 3D Slicer software; and (ii) iBraiN2 and iBraiNEEG2, which are mobile based applications with functional connectivity networks, based in network-level parcellation (Yeo et al., 2011).

iBraiN2 is a tool for academic use that shows the three-dimensional position of each functional connectivity network in the cerebral cortex. Using a transparent brain, it is possible to see the position of each region in its connectivity network or the relative position of each network. Although this 3D representation only shows a fixed dataset, is useful for academic purposes for neurologists, neurosurgeons, radiologists, medical students, neuroscientists and researchers in neuroimaging, among other professionals, since it allows them to easily understand in an interactive way the anatomy and distribution of the brain’s functional networks, because it shows the standard functional connectivity networks (Yeo et al., 2011).

iBraiNEEG2 shows the relative position of the 10–20 EEG electrodes with respect to the functional connectivity networks. This application helps the EEG analysis regarding standard functional connectivity networks (Yeo et al., 2011) and combines EEG data and functional connectivity obtained with fMRI to support the analysis of these techniques and research into diseases such as epilepsy. For example, with the EEG data of the epileptic focus of a patient, it could be possible to get the RS-fMRI functional connectivity network that is affected.

Should the scientific community adopt a brain atlas as a functional connectivity gold standard, future updates of the applications would greatly benefit from the use of such atlas. A very relevant candidate for this standard would be a recently published brain atlas (Glasser et al., 2016) that uses multi-modal magnetic resonance images from the Human Connectome Project (HCP).

In conclusion, the applications for mobile devices shown here are useful for education and training purposes (medical professionals) in functional connectivity related topics and EEG analysis regarding standard functional connectivity networks. Also, the advantage of applications for mobile devices is associated with their portability and availability.

## Author Contributions

GMR: article writing, creation of the figures, mobile applications design, mobile applications testing (Android), creation and optimization of functional connectivity networks mesh. JAF: mobile applications design and programming, mobile applications testing (Android). MG: mobile applications design, mobile applications testing (iOS), article review and correction.

## Conflict of Interest Statement

The authors declare that the research was conducted in the absence of any commercial or financial relationships that could be construed as a potential conflict of interest.

## Footnotes

^1^http://www.slicer.org

^2^https://surfer.nmr.mgh.harvard.edu/fswiki/CorticalParcellation_Yeo2011

^3^http://meshlab.sourceforge.net

^4^http://www.unity.com

^5^http://www.monodevelop.com

^6^http://www.blender.org

^7^http://www.gimp.org

^8^http://www.apple.com

## References

[B1] Cignoni P. Callieri M. Corsini M. Dellepiane M. Ganovelli F. Ranzuglia G. (2008a). “Meshlab: an open-source mesh processing tool,” in Proceedings of the 6th Eurographics Italian Chapter Conference (Pisa, Italy: Eurographics), 129–136.

[B2] Cignoni P. Corsini M. Ranzuglia G. (2008b). Meshlab: an open-source 3d mesh processing system. Ercim News 2008, 47–48.

[B3] Craddock R. C. James G. A. Holtzheimer P. E. III Hu X. P. Mayberg H. S. (2012). A whole brain fMRI atlas generated via spatially constrained spectral clustering. Hum. Brain Mapp. 33, 1914–1928. 10.1002/hbm.21333 21769991 PMC3838923

[B4] de la Iglesia-Vayá M. Molina-Mateo J. Escarti-Fabra M. J. Kanaan A. S. Martí-Bonmatí L. (2013). “Brain connections—resting state fmri functional connectivity,” in Novel Frontiers of Advanced Neuroimaging ed. Fountas K. N. (InTech). Available online at: http://www.intechopen.com/books/novel-frontiers-of-advanced-neuroimaging/brain-connections-resting-state-fmri-functional-connectivity

[B5] Gering D. T. Nabavi A. Kikinis R. Grimson W. E. L. Hata N. Everett P. (1999). An integrated visualization system for surgical planning and guidance using image fusion and interventional imaging. Int. Conf. Med. Image Comput. Comput. Assist. Interv. 2, 809–819. 10.1007/10704282_88

[B6] Glasser M. F. Coalson T. S. Robinson E. C. Hacker C. D. Harwell J. Yacoub E.. (2016). A multi-modal parcellation of human cerebral cortex. Nature 536, 171–178. 10.1038/nature18933 27437579 PMC4990127

[B7] Greicius M. (2008). Resting-state functional connectivity in neuropsychiatric disorders. Curr. Opin. Neurol. 21, 424–430. 10.1097/wco.0b013e328306f2c5 18607202

[B8] IDC Research, Inc. (2015). Smartphone OS market share, 2015 Q2. Available online at: http://www.idc.com/prodserv/smartphone-os-market-share.jsp

[B9] Jasper H. H. (1958). The ten-twenty electrode system of the International federation. Electroencephalogr. Clin. Neurophysiol. 10, 371–375. 10590970

[B10] Klem G. H. Lüders H. O. Jasper H. H. Elger C. (1999). The ten-twenty electrode system of the International federation. The International federation of clinical neurophysiology. Electroencephalogr. Clin. Neurophysiol. Suppl. 52, 3–6. 10590970

[B11] Lorensen W. E. Cline H. E. (1987). “Marching cubes: a high resolution 3D surface construction algorithm,” in SIGGRAPH ’87 Proceedings of the 14th Annual Conference on Computer Graphics and Interactive Techniques (New York, NY: ACM SIGGRAPH Computer Graphics) 21, 163–169.

[B12] Mandal P. K. Mahajan R. Dinov I. D. (2012). Structural brain atlases: design, rationale and applications in normal and pathological cohorts. J. Alzheimers Dis. 31, S169–S188. 10.3233/JAD-2012-120412 22647262 PMC4324755

[B13] Margulies D. S. Böttger J. Watanabe A. Gorgolewski K. J. (2013). Visualizing the human connectome. Neuroimage 80, 445–461. 10.1016/j.neuroimage.2013.04.111 23660027

[B15] Mazziotta J. C. Toga A. W. Evans A. Fox P. Lancaster J. (1995). A probabilistic atlas of the human brain: theory and rationale for its development. The International consortium for brain mapping (ICBM). Neuroimage 2, 89–101. 10.1006/nimg.1995.1012 9343592

[B14] Mazziotta J. Toga A. Evans A. Fox P. Lancaster J. Zilles K.. (2001). A probabilistic atlas and reference system for the human brain: International consortium for brain mapping (ICBM). Philos. Trans. R. Soc. Lond. B Biol. Sci. 356, 1293–1322. 10.1098/rstb.2001.0915 11545704 PMC1088516

[B16] Nuwer M. R. Comi G. Emerson R. Fuglsang-Frederiksen A. Guérit J. M. Hinrichs H.. (1998). IFCN standards for digital recording of clinical EEG. International federation of clinical neurophysiology. Electroencephalogr. Clin. Neurophysiol. 106, 259–261. 10.1016/s0013-4694(97)00106-5 9743285

[B17] Payne K. B. Wharrad H. Watts K. (2012). Smartphone and medical related App use among medical students and junior doctors in the United Kingdom (UK): a regional survey. BMC Med. Inform. Decis. Mak. 12:121. 10.1186/1472-6947-12-121 23110712 PMC3504572

[B18] Pieper S. Halle M. Kikinis R. (2004). “3D SLICER,” in *Proceedings of the 1st IEEE International Symposium on Biomedical Imaging*: from Nano to Macro *vol. 1; 2004*, (Arlington, VA), 632–635.

[B19] Pieper S. Lorensen B. Schroeder W. Kikinis R. (2006). “The NA-MIC kit: ITK, VTK, pipelines, grids and 3D slicer as an open platform for the medical image computing community,” in *Proceedings of the 3rd IEEE International Symposium on Biomedical Imaging*: from Nano to Macro (Arlington, VA), 698–701.

[B20] Proal E. Alvarez-Segura M. de la Iglesia-Vayá M. Martí-Bonmatí L. Castellanos F. X. Spanish Resting State Network. (2011). Functional cerebral activity in a state of rest: connectivity networks. Rev. Neurol. 52, S3–S10. 21365601 PMC4418791

[B21] Raichle M. E. MacLeod A. M. Snyder A. Z. Powers W. J. Gusnard D. A. Shulman G. L. (2001). A default mode of brain function. Proc. Natl. Acad. Sci. U S A 98, 676–682. 10.1073/pnas.98.2.676 11209064 PMC14647

[B22] Rojas G. M. Gálvez M. (2013). “Functional connectivity networks obtained using 10–20 EEG and 7 standard functional networks,” in *19th Annual Meeting of the Organization for Human Brain Mapping*, Seattle, WA.

[B23] Rojas G. M. Gálvez M. Vega Potler N. Craddock R. C. Margulies D. S. Castellanos F. X.. (2014). Stereoscopic three-dimensional visualization applied to multimodal brain images: clinical applications and a functional connectivity atlas. Front. Neurosci. 8:328. 10.3389/fnins.2014.00328 25414626 PMC4222226

[B24] Ventola C. L. (2014). Mobile devices and apps for health care professionals: uses and benefits. P T 39, 356–364. 24883008 PMC4029126

[B25] Vollmer J. Mencl R. Müller H. (1999). Improved Laplacian smoothing of noisy surface meshes. Comput. Graph. Forum 18, 131–138. 10.1111/1467-8659.00334

[B26] Wilson S. (2013). Rising tide. Exploring pathways to growth in the mobile semiconductor industry. Available online at: http://dupress.com/articles/rising-tide-exploring-pathways-to-growth-in-the-mobile-semiconductor-industry

[B27] Yeo B. T. Krienen F. M. Sepulcre J. Sabuncu M. R. Lashkari D. Hollinshead M.. (2011). The organization of the human cerebral cortex estimated by intrinsic functional connectivity. J. Neurophysiol. 106, 1125–1165. 10.1152/jn.00338.2011 21653723 PMC3174820

